# A hybrid method for inversion of 3D DC resistivity logging measurements

**DOI:** 10.1007/s11047-014-9440-y

**Published:** 2014-07-24

**Authors:** Ewa Gajda-Zagórska, Robert Schaefer, Maciej Smołka, Maciej Paszyński, David Pardo

**Affiliations:** 1 AGH University of Science and Technology, Krakow, Poland; 2 BCAM and Ikerbasque, University of the Basque Country UPV/EHU, Bilbao, Spain

**Keywords:** Hierarchic genetic strategy, Inverse problem, Hybrid method

## Abstract

This paper focuses on the application of *hp* hierarchic genetic strategy (*hp*–HGS) for solution of a challenging problem, the inversion of 3D direct current (DC) resistivity logging measurements. The problem under consideration has been formulated as the global optimization one, for which the objective function (misfit between computed and reference data) exhibits multiple minima. In this paper, we consider the extension of the *hp*–HGS strategy, namely we couple the *hp*–HGS algorithm with a gradient based optimization method for a local search. Forward simulations are performed with a self-adaptive *hp* finite element method, *hp*–FEM. The computational cost of misfit evaluation by *hp*–FEM depends strongly on the assumed accuracy. This accuracy is adapted to the tree of populations generated by the *hp*–HGS algorithm, which makes the global phase significantly cheaper. Moreover, tree structure of demes as well as branch reduction and conditional sprouting mechanism reduces the number of expensive local searches up to the number of minima to be recognized. The common (direct and inverse) accuracy control, crucial for the* hp*–HGS efficiency, has been motivated by precise mathematical considerations. Numerical results demonstrate the suitability of the proposed method for the inversion of 3D DC resistivity logging measurements.

## Introduction

To estimate the subsurface electrical properties, it is customary to record resistivity measurements using logging instruments that move along a borehole axis. These instruments are equipped with several transmitter electrodes, whose emitted signal is recorded by the receiver electrodes that are also located along the tool.

Logging instruments are designed in such a way that the voltage combination measured at receivers depends on the formation’s electrical conductivity. Thus, logging instruments are intended to estimate properties (electrical conductivity) of the sub-surface material. The ultimate goal is to identify and characterize hydrocarbon (oil and gas) bearing formations. In order to design better logging instruments as well as for improving the interpretation of the recorded measurements, computer simulations of resistivity logging measurements are essential and widely used in many geophysical applications such as hydrocarbon (oil and gas) exploration.

In this paper, we focus on borehole logging devices operating of very low frequencies (close to zero), which are numerically modeled as zero-frequency direct current (DC). We perform simulations of 3D resistivity measurements in deviated wells, with an angle between the borehole and formation layers below 90°. We consider two types of problems: forward and inverse. The former consists of finding the voltage for a certain position of transmitter and receiver electrodes given known resistivities of formation layers. A series of forward problems for consecutive positions of electrodes provides a sequence of solutions forming a *logging curve*. In the inverse problem, formalized as a global optimization one, we are given a reference logging curve and seek for parameters (resistivities of formation layers) that would result in a similar curve.

There exist a plethora of numerical simulation methods developed to improve the simulation of forward resistivity measurements (i.e. Avdeev et al. 2002; Davydycheva et al. 2003; Druskin et al. 1999; Newman and Alumbaugh 2002; Pardo et al. 2008; Wang and Fang 2001; Zhang et al. 1995; Wang and Signorelli 2004). Since each simulation requires solution of a partial differential equation in 3D, the computational cost associated to the solution of a forward problem is elevated. In order to minimize such cost without compromising the accuracy, we employ a forward solver (Pardo et al. 2008) based on a combination of a Fourier series expansion in a non-orthogonal system of coordinates with a 2D self-adaptive *hp* goal-oriented finite element method (*hp*–FEM) (see Pardo et al. 2006a, c, 2007). This Fourier-finite-element method was formulated and applied to direct and alternating current resistivity logging problems, and it enabled fast and accurate simulations of resistivity measurements in deviated wells.

When dealing with inverse problems, several challenges appear. First, these problems are often ill-conditioned and a small change in parameters may cause a huge difference in results. Moreover, they may have a non-unique solution. Additionally, the appearance of multiple minima (multimodality) may make the search of the global optimum difficult.

The inverse problem under consideration (inversion of 3D DC resistivity logging measurement) is much less sensitive to the conductivities of layers saturated by oil or gas than to the conductivities of others surrounding layers e.g., rock, sand (see Szeliga 2013). Moreover, the measured response from layers saturated by oil or gas has a remarkable dispersion, which is frequently reported by practitioners. As a result, we may expect more than one inverse solution outlining the range of conductivities of such layers.

Several strategies for the inversion of resistivity logging measurements use the convex optimization methods only (e.g., Abubakar and Berg 2000; Abubakar et al. 2006; Zhang et al. 1995). Unfortunately, they do not deliver guarantee of finding all solutions. Another possibility is to use stochastic, evolutionary methods (e.g., Burczyński and Osyczka 2004; Kern et al. 2004; Pan et al. 2011), but their applicability is restricted by a huge computational cost and moderate accuracy. The computational cost problem may be partially overcome by using two-phase strategies in which a stochastic algorithm is used as a preprocessor (the global phase) for selecting starting points of convex optimization processes (the local phase) (e.g., Schaefer et al. 2004; Törn 1975).

The main goal of this paper is to introduce the two-phase strategy that offers the asymptotic guarantee of success (see e.g., Horst and Pardalos 1995) and allows for dealing with multimodality, delivering a high final accuracy with an exceptionally low computational cost for inversion of 3D DC resistivity logging measurements.

The global phase is performed by the dynamically adjustable Hierarchic Genetic Strategy *hp*–HGS (Schaefer and Kołodziej 2003).

This strategy develops a tree of dependent demes. The root-deme performs the most chaotic search with low accuracy. Along with going deeper in the tree, the search becomes more local and accurate. The strategy starts with the root-deme only. After a number of epochs (the metaepoch), the best individual is selected as a seed of the child-deme. Sprouting new demes is repeated concurrently for root and all branches excluding leaves. It is performed conditionally, if there is room for new deme among existing ones at the particular level of the *hp*–HGS tree (the distance between centers of existing demes and a seed of a new deme is sufficiently large). Moreover, child-demes at each level are periodically checked, and redundant demes are reduced (joined and commonly selected). Evolutionary processes in branches and leaves are stopped if no progress is observed. The whole strategy is stopped if a sufficient number of well fitted leaves are obtained. Both, binary and real-valued encoding simple genetic algorithm (SGA) and simple evolutionary algorithm (SEA) are utilized.

The local phase consists of running local gradient method starting at the satisfactory fitted individuals, at most one per one leaf-deme. In particular, we utilize the Broyden–Fletcher–Goldfarb–Shanno (BFGS) algorithm, a quasi-Newton method utilizing the approximation to the Hessian matrix.

The HGS structure results in much less total fitness evaluations than a single population algorithm searching with the maximum accuracy (see e.g., Schaefer and Kołodziej 2003; Wierzba et al. 2003). Only the root-deme searches continuously with a large number of individuals. Branches and leaves are small demes invoked only in the promising regions found by they parental demes and quickly terminated, just after they stop to search effectively.

Next, the conditional sprouting and redundancy reduction among child demes significantly decreases a number of fitness evaluations. Moreover, these mechanisms allow for concurrent identification of separate basins of attractions by separate well fitted leaf-demes. The target accuracy in the global phase utilized by leaf-demes should not be so high, only enough to separate basins of attractions of different minimizers.

A huge cost reduction is caused by the scaling of the fitness evaluation error. Forward simulations are performed with a self-adaptive *hp* goal-oriented Finite Element Method. The computational cost of misfit evaluation by *hp*–FEM depends strongly on the assumed accuracy. This accuracy is adapted to the inverse error at the particular level of the population tree generated by *hp*–HGS, which makes the global phase cheaper. The necessary mathematical motivation will be delivered in Sect. 6 and preceding Sects. 2–4.

Only the necessary minimum number of local searches is activated for finding all minimizers with high accuracy. The local gradient searches are expensive in case of numerical gradient evaluation, which is necessary if, for some reasons it can not be obtained analytically (e.g., misfit irregularity or lack of its algebraic formula). Such strategy outperforms the multistart with a uniform sampling of starting points (Törn 1975).

Contrary to traditional inversion algorithms that produce a unique solution to the problem, our hybrid strategy delivers multiple solutions, which enables an expert on the field to determine the best possible solution as well as the uncertainty level.

The idea of *hp*–HGS was introduced in 2007 Paszyński et al. (2007). Asymptotic guarantee of success of HGS was proved in Schaefer and Kołodziej (2003). Analysis of the asymptotic guarantee of success and the computational cost reduction with respect to the single- and multi-deme strategies without the common scaling of forward and inverse errors is performed in Schaefer and Barabasz (2008). The papers Barabasz et al. (2009, 2011b), show the theory necessary for applying *hp*–HGS to the inverse, parameter problems in heat flow. They contain also the computational examples of finding multiple solutions by *hp*–HGS. Similar results related to the *hp*–HGS application to the inverse, parametric elasticity problems were presented in Barabasz et al. (2011a).

The novelty of this paper consists of applying the two-phase strategy combining *hp*–HGS and local methods for the inversion of 3D DC resistivity logging measurements. Moreover, all mathematical derivations leading to the forward and inverse error relationship necessary for the strategy verification are new. All presented simulations showing the strategy in action have not been published before. Additionally, we compare results obtained by our proposed *hp*–HGS–BFGS method with two state-of-the-art methods frequently applied for solving ill-posed multimodal problems, showing the superior performance of the proposed method.

The paper is organized as follows. In Sect. 2, we describe our model forward problem, which is governed by the conductive media equation. We also introduce a dual forward problem. Sect. 3 outlines the application of *hp* Finite Element strategy for forward simulations. In the next sections, we consider inverse problems. A general introduction to this topic is provided in Sect. 4. Section 5 describes the Hierarchic Genetic Strategy with binary and real-number encodings. Then, we discuss the relation between approximate forward and inverse solutions errors in Sect. 6. Section 7 analyzes the *hp*–HGS strategy for solving dual inverse problems.

Section 8 discusses briefly the advantages of the proposed hybridization and compares its features with other stochastic strategies. Section 9 describes the numerical inversion of resistivity measurements obtained using the hybrid strategy. In Sect. 10, we compare our results with simulations obtained with two state-of-the-art global optimization methods: the Simple Evolutionary Algorithm and the multistart method.

Finally, conclusions are outlined in Sect. 11.

## Forward problems

### DC conductive media equation

The direct current flow in the continuum 3D conductor is governed by the so called *conductive media equation*

1 $$\begin{aligned} \displaystyle {\varvec{\nabla }}\cdot ({\varvec{\sigma }}{\varvec{\nabla }}u ) = -{\varvec{\nabla }}\cdot \mathbf{J}^{imp} \, , \end{aligned}$$

where ${\varvec{\sigma }}$ is the conductivity tensor field, $\mathbf{J}^{imp}$ represents the prescribed, impressed electric current source, and $u$ is the scalar electric potential.

**Fig. 1:**
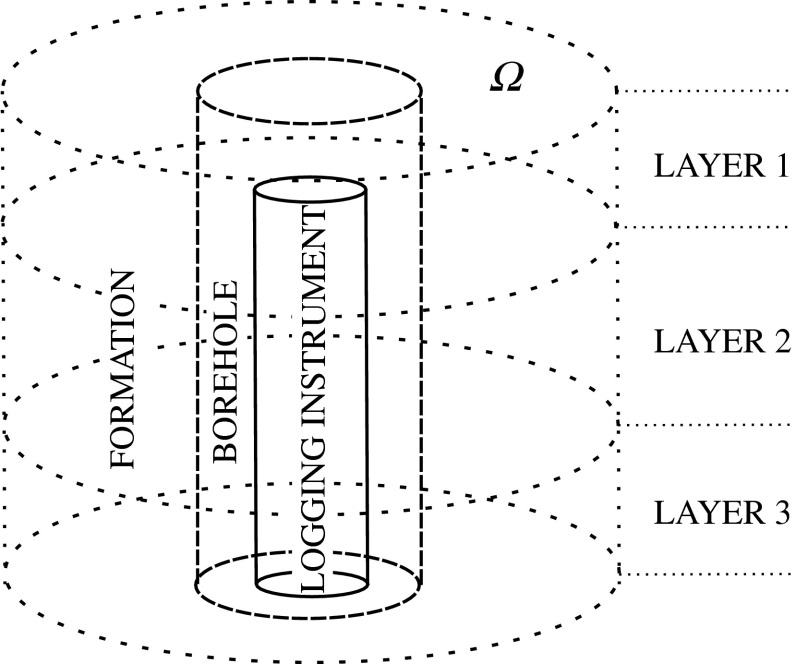
3D geometry of a logging instrument in a *vertical borehole* penetrating three dipping layers

We are looking for solutions to (1) in the domain $\varOmega \in {\mathbb {R}}^3$ being a 3D cylinder surrounding the borehole (see Fig. 1). Notice that such $\varOmega$ is a simply connected bounded domain with Lipschitz boundary. We assume Dirichlet and Neumann boundary conditions $u_D$ and $h$ on the separate nonintersecting parts $\Gamma _D$, $\Gamma _N$ of $\partial \varOmega$, respectively.

Multiplying test function $v \in H_D^1(\varOmega )=\{ u \in H^1(\varOmega ) : \, u|_{\Gamma _D}=0 \}$ by equation (1), and integrating by parts over the domain $\varOmega$, we obtain the following variational formulation:

2 $$ \begin{aligned} \left\{ \begin{array}{l} \hbox {Find } u \in u_{D} + H_D^1(\varOmega ) \hbox { such that: }\\ \begin{array}{lll} ( {\varvec{\sigma }}{\varvec{\nabla }}u {\varvec{\nabla }}v )_{L^2(\varOmega )}  = ( {\varvec{\nabla }}\cdot \mathbf{J}^{imp} \,,\, v )_{L^2(\varOmega )}\\+( h \,,\, v )_{L^2(\Gamma _N)}\\ \end{array}\\ \forall v \in H_D^1(\varOmega ), \end{array}\right. \end{aligned} $$

where $u_D \in H^1(\varOmega )$ is a lift of the essential Dirichlet data $u_D$ (denoted with the same symbol), $\displaystyle h={\varvec{\sigma }}{\varvec{\nabla }}u \cdot \mathbf{n}$ is a prescribed flux on $\Gamma _N$, $\mathbf{n}$ is the unit normal outward (with respect to $\varOmega$) vector, and $u|_{\Gamma _D}=0$ is understood in the sense of traces.

We assume that

3 $$\begin{aligned}&\mathbf{J}^{imp} \in H(div;\varOmega ), \; \; h \in H^1(\partial \varOmega ),\end{aligned}$$

4 $$\begin{aligned}&{\varvec{\sigma }}_{i,j} \in L^\infty (\varOmega ), \; \; \; i,j = 1,2,3, \quad \text{ i.e. } \exists M > 0; \; \; |{\varvec{\sigma }}_{i,j}| \le M, \; \; i,j = 1,2,3,  \end{aligned}$$

almost everywhere in *Ω* , and

5 $$ \begin{aligned} \begin{array}{l} \exists c_0 > 0, \; \forall \xi \in {\mathbb {R}}^3;\\ \sum \limits_{i,j=1,2,3} \, {\varvec{\sigma }}_{i,j} \, \xi _i \, \xi _j \; \ge \; c_0 \sum \limits _{i=1,2,3} \; \xi _i^2,  \end{array} \end{aligned} $$

almost everywhere in *Ω*.

In the sequel, we shall consider only the case in which ${\varvec{\sigma }}$ is a scalar field i.e. ${\varvec{\sigma }}_{i,j} = \sigma \, \delta _{i,j}$ where $\sigma$ is a scalar conductivity. Instead of (4), (5) we assume that:

6 $$\begin{aligned} \begin{array}{l} \exists c_0, M; \; 0 < c_0 < M < +\infty ,\\ c_0 \le \sigma \le M \; \text{ almost } \text{ everywhere } \text{ in } \; \varOmega . \end{array} \end{aligned}$$

Of course (6) implies that $\sigma \in L^\infty (\varOmega )$. Moreover, we set $u_D=0$ and $\Gamma _N = \emptyset$ so (2) is reduced to the form:

7 $$\begin{aligned} \left\{ \begin{array}{l} \text{ Find } u \in H_0^1(\varOmega ) \hbox { such that: }\\ \displaystyle\; ( \sigma {\varvec{\nabla }}u \,,\, {\varvec{\nabla }}v )_{L^2(\varOmega )} = ( q \,,\, v )_{L^2(\varOmega )} \; \; \; \; \forall v \in H_0^1(\varOmega ), \end{array}\right. \end{aligned}$$

where ${\varvec{\nabla }}\cdot \mathbf{J}^{imp} = q \in L^2(\varOmega )$ is the intensity of the “current source” imposed by the probe. The above relation (7) will be called the *primal forward problem* of DC conduction.

For the case of deviated wells (below $90^\circ$) in a horizontally stratified layered media, we employ the *hp*–Fourier finite element method described in Pardo et al. (2008). This method performs a non-orthogonal change of coordinates followed by a Fourier series expansion in the azimuthal direction. Using that technique, we obtain fast and accurate forward simulations of 3D resistivity logging measurements in deviated wells.

### Abstract formulation

Let us rewrite (7) into a more convenient abstract form. First, we introduce the trilinear form:

8 $$\begin{aligned} \begin{array}{l} b: L^\infty (\varOmega ) \times H_0^1(\varOmega )^2 \ni (\sigma , u, v) \rightarrow \\ b(\sigma ; u, v) = ( \sigma {\varvec{\nabla }}u \,,\, {\varvec{\nabla }}v )_{L^2(\varOmega )} \in {\mathbb {R}} \end{array} \end{aligned}$$

Assumption (6) allows to define the family of operators

9 $$\begin{aligned} B: L^\infty (\varOmega ) \times H_0^1(\varOmega ) \ni (\sigma ; u) \rightarrow B(\sigma ; u) \in H^{-1}(\varOmega ) \end{aligned}$$

indexed by $\sigma \in L^\infty (\varOmega )$, so that

10 $$\begin{aligned} \begin{array}{l} <B(\sigma ; u), v> \, = \, b(\sigma ; u, v),\\ \forall u, v \in H_0^1(\varOmega ),\; \forall \sigma \in L^\infty (\varOmega ), \end{array} \end{aligned}$$

where $< \cdot , \cdot >$ denotes the parity between $H_0^1(\varOmega )$ and $H^{-1}(\varOmega )$ (see Denkowski et al. (2003a, b), for details). Moreover $q \in L^2(\varOmega )$ allows for defining the linear, continuous functional $F \in H^{-1}(\varOmega )$ so that

11 $$\begin{aligned} F: H_0^1(\varOmega ) \ni v \rightarrow ( q \,,\, v )_{L^2(\varOmega )} \in {\mathbb {R}}. \end{aligned}$$

We will later denote the solution to the primary forward problem as $u(\sigma )$ in order to highlight its dependence on the assumed conductivity field $\sigma$.

The family of primal forward problems indexed by $\sigma \in L^\infty (\varOmega )$ may be written as follows:

12 $$\begin{aligned} \left\{ \begin{array}{l} \text{ Find } u(\sigma ) \in H_0^1(\varOmega ) \hbox {such that}: \\ \displaystyle\; b(\sigma ; u(\sigma ), v) = F(v) \; \; \forall v \in H_0^1(\varOmega ), \end{array}\right. \end{aligned}$$

or as the family of equations in $H^{-1}(\varOmega )$

13 $$\begin{aligned} \left\{ \begin{array}{l} \text{ Find } u(\sigma ) \in H_0^1(\varOmega ) \hbox { such that:}\\ \displaystyle\; B(\sigma ; u(\sigma )) = F. \end{array}\right. \end{aligned}$$

### Dual forward problem

The crucial aspect of the solution $u(\sigma )$ to the primal forward problem (2) will be its mean value over the subdomain $\varOmega _P \subset \varOmega$ occupied by the receiver part of the probe. We will define the indicator functional:

14 $$\begin{aligned} H_0^1(\varOmega ) \ni v \rightarrow \; < Q, v > \; = \frac{1}{\text{ meas }(\varOmega _P)}\int \limits _{\varOmega _P} v \, dx \in {\mathbb {R}}. \end{aligned}$$

Obviously $$Q \in H^{-1}(\varOmega )$$ because

$$ \begin{aligned} \begin{array}{lll} \left| \frac{1}{\text{ meas }(\varOmega _P)}\int \limits _{\varOmega _P} v \, dx \right | &=& \frac{1}{\text{ meas } (\varOmega _P)} \left| \int \limits _{\varOmega } \chi _{\varOmega _P} \, v \, dx \right| \\ & \le & \frac{1}{\text{ meas } (\varOmega _P)} \left\| \chi _{\varOmega _P} \right\| _{L^2(\varOmega )} \, \left\| v \right\| _{L^2(\varOmega )}\\ & \le & C \, \left\| v \right\| _{H^1_0(\varOmega )}, \end{array} \end{aligned} $$

where *C* contains the norm equivalence constant on $H^1_0(\varOmega )$. The functional *Q* is sometimes called the *quantity of interest* (see Oden and Prudhomme 2001).

Now we are ready to define the family of *dual forward problems*

15 $$\left\{ \begin{array}{l} \text{Find } G(\sigma ) \in H_0^1(\varOmega ) \hbox {such that}: \\  b(\sigma ; G(\sigma ), w) = Q(w) \; \; \forall w \in H_0^1(\varOmega ), \end{array}\right. $$

or as the family of equations in $H^{-1}(\varOmega )$

16 $$\begin{aligned} \left\{ \begin{array}{l} \text{Find } G(\sigma ) \in H_0^1(\varOmega ) \hbox {such that}: \\  B(\sigma ; G(\sigma )) = Q, \end{array}\right. \end{aligned}$$

indexed by $$\sigma \in L^\infty (\varOmega )$$.

### Basic features of forward problems

It is easy to observe that $b(\sigma ; \cdot , \cdot )$ is symmetric, and Lipschitz continuous in both variables with the constant $M$, and coercive with the constant $c_0$ uniformly with $\sigma$ satisfying (6).

#### *Remark 1*

Given all above assumptions, both forward problems (primal and dual ones) (12), (15) have the unique solutions $u(\sigma )$, $G(\sigma )$ (see Denkowski et al. (2003a, b), for details) for each fixed $\sigma$ satisfying (6). Moreover, the solution to the primal forward problem $u(\sigma )$ depends continuously on $q$ (in $L^2(\varOmega )$ and $H_0^1(\varOmega )$ topologies) while $G(\sigma )$ on $Q$ (in $H^{-1}(\varOmega )$ and $H_0^1(\varOmega )$ topologies).

#### *Remark 2*

Since $b(\sigma ; \cdot , \cdot )$ is symmetric, then (15) may take a form:

17 $$\begin{aligned} \left\{ \begin{array}{l} \text{Find } G(\sigma ) \in H_0^1(\varOmega ) \hbox { such that: }\\ \displaystyle b(\sigma ; w, G(\sigma )) = Q(w) \; \; \forall w \in H_0^1(\varOmega ). \end{array}\right. \end{aligned}$$

Because $H_0^1(\varOmega )$ is reflexive (i.e. $(H_0^1(\varOmega ))''$ is isomorphic with $H_0^1(\varOmega )$), we may associate the solution $G(\sigma )$ to (17) to an element of $(H_0^1(\varOmega ))''$ such that, in particular

18 $$\begin{aligned} < G(\sigma ), F > \; = \; F(G(\sigma )) \; = \; < Q, u(\sigma )>, \end{aligned}$$

where the angle brackets at the left-hand side denote the parity between $(H_0^1(\varOmega ))''$ and $H^{-1}(\varOmega )$, while the parity between $H^{-1}(\varOmega )$ and $H_0^1(\varOmega )$ is denoted by the angle brackets at the right-hand side. $G(\sigma )$ might be then interpreted as the functional that returns the quantity of interest associated with the solution $u(\sigma )$ to the primal forward problem (13) obtained for the right-hand side $F$ being its argument.

#### *Remark 3*

Coupling (18) with (12) and (15), we obtain

19 $$\begin{aligned} F(G(\sigma )) = b(\sigma ; u(\sigma ), G(\sigma )) = Q(u(\sigma )) \end{aligned}$$

or using the parity convention

20 $$\begin{aligned} <G(\sigma ), F> = <B(\sigma ; u(\sigma )), G(\sigma )> = <Q, u(\sigma )>. \end{aligned}$$

#### **Lemma 1**

*The solution to the primal forward problem* (12)* depends Lipschitz-continuously on the parameter* $\sigma$ *i.e*.

21 $$\begin{aligned} \exists C > 0; \, \left\| u(\sigma ^1) - u(\sigma ^2) \right\| _{H^1_0(\varOmega )} \le C \left\| \sigma ^1 - \sigma ^2 \right\| _{L^\infty (\varOmega )}. \end{aligned}$$

#### *Proof*:

 We will follow strictly the ideas of the proof of Theorem 3.1 from our earlier paper Barabasz et al. (2011b). Let us denote for convenience $u_1 = u(\sigma ^1), u_2 = u(\sigma ^2)$, two solutions to the primal forward problem (12). We have $b(\sigma ^1; u_1, v) = b(\sigma ^2; u_2, v) = F(v) \; \forall v \in H_0^1(\varOmega )$. Then:

$$ b(\sigma^1; {u_1} - {u_2,v}) =({\sigma^1} {\varvec{\nabla}}({u_1} - {u_2}), {{\varvec{\nabla}}}v )_{{L^2}(\varOmega)} = ({\sigma^1} {\varvec{\nabla }}u_1 - {\sigma^1} {\varvec{\nabla}}{u_2} + {\sigma^2} {\varvec{\nabla }}{u_2} - {\sigma^2} {\varvec{\nabla }}{u_2}, {\varvec{\nabla}}v)_{L^2(\varOmega)} = -(({\sigma^1} - {\sigma^2}) {\varvec{\nabla }}{u_2}, {\varvec{\nabla}}v)_{{L^2}(\varOmega )} = -b({\sigma^1} - {\sigma^2}; {u_2,v}).$$

Then,

$$\begin{aligned} \begin{array}{lll} c_0 \left\| u_1 - u_2 \right\| ^2_{H^1_0(\varOmega )} &\le &| b(\sigma ^1; u_1 - u_2, v) |   = | b(\sigma ^1 - \sigma ^2; u_2, v) |. \end{array} \end{aligned}$$

Using Hölder inequality, we obtain:

$$\begin{aligned} \begin{array}{l}c_0 \left\| u_1 - u_2 \right\| ^2_{H^1_0(\varOmega )}\\ \quad\le \left\| \sigma ^1 - \sigma ^2 \right\| _{L^\infty (\varOmega )} \; \left\| u_2 \right\| _{H^1_0(\varOmega )} \; \left\| u_1 - u_2 \right\| _{H^1_0(\varOmega )}\\ \quad\le \frac{1}{c_0} \left\| F \right\| _{H^{-1}(\varOmega )} \; \left\| \sigma ^1 - \sigma ^2 \right\| _{L^\infty (\varOmega )} \; \left\| u_1 - u_2 \right\| _{H^1_0(\varOmega )}. \end{array} \end{aligned}$$

Finally, $$\left\| u_1 - u_2 \right\| _{H^1_0(\varOmega )} \le C \, \left\| \sigma ^1 - \sigma ^2 \right\| _{L^\infty (\varOmega )}$$, where $$C = \frac{1}{(c_0)^2} \left\| F \right\| _{H^{-1}(\varOmega )}$$. $$\square $$

### Galerkin solutions

Let us study now the Galerkin solutions to both primal and dual problems (12), (13), (15), (16). We introduce the sequence $\{X_i\}_{i=1}^{+\infty }$ of subspaces of $H_0^1(\varOmega )$ so that $\overline{X}_i \subset X_{i+1}$, $i = 1,2,3, \ldots$ and $\text{ dim }(X_i) = n_i < +\infty$, $n_{i+1} > n_i$. Moreover, $\forall u \in H_0^1(\varOmega )$

22 $$\begin{aligned} \text{ lim }_{i \rightarrow +\infty } \left\{ \text{ inf }_{u_i \in X_i} \left\| u_i - u \right\| _{H_0^1(\varOmega )} \right\} = 0, \end{aligned}$$

which implies that $\overline{\bigcup _{i=1}^{+\infty } X_i} = H_0^1(\varOmega )$.

Let us define the approximate family of *Galerkin primal forward problems*:

23 $$\begin{aligned} \left\{ \begin{array}{l} \text{ Find }u_i(\sigma ) \in X_i \hbox { such that:}\\\displaystyle \;b(\sigma ; u_i(\sigma ), v) = F(v) \; \; \forall v \in X_i, \end{array}\right. \end{aligned}$$

and the *Galerkin dual forward problem*:

24 $$\begin{aligned} \left\{ \begin{array}{l} \text{ Find }G_i(\sigma ) \in X_i \hbox { such that:}\\\displaystyle \;b(\sigma; w, G_i(\sigma )) = Q(w) \; \; \forall w \in X_i,\end{array}\right.\end{aligned}$$

where now $b:L^\infty (\varOmega ) \times X_i \times X_i \rightarrow {\mathbb {R}}$ is the restriction of the bilinear form $b$, and $F:X_i \rightarrow {\mathbb {R}}$, $Q:X_i \rightarrow {\mathbb {R}}$ are the restrictions of the right-hand side functionals. For the sake of simplicity, we do not introduce new descriptions for these restrictions. Their correct meaning will be determined by the context.

The assumed features of $b$, $F$, and $Q$ imply that

25 $$\begin{aligned} \begin{array}{l} \left\| u_i(\sigma ) - u(\sigma ) \right\| _{H_0^1(\varOmega )} \rightarrow 0,\\ \left\| G_i(\sigma ) - G(\sigma ) \right\| _{H_0^1(\varOmega )} \rightarrow 0\\ \text{ for } \; \; i \rightarrow +\infty , \end{array} \end{aligned}$$

where $u(\sigma ), G(\sigma )$ are the exact solutions to the primal and dual forward problems (12), (13), (15), (16) (see e.g. Ciarlet (1978)). Furthermore, Remark 3 implies that

26 $$\begin{aligned} \begin{array}{l} \forall i = 1, 2, 3, \dots \\ F(G_i(\sigma )) = b(\sigma ; u_i(\sigma ), G_i(\sigma )) = Q(u_i(\sigma )), \end{array} \end{aligned}$$

where $u_i(\sigma ), G_i(\sigma ) \in X_i$ are the corresponding solutions to the Galerkin primal and dual forward problems, respectively.

Let us prove a lemma that is convenient for future error estimations.

#### **Lemma 2**

*Let* $u_i(\sigma ), u_j(\sigma ), \; i > j$ *be two consecutive solutions of the Galerkin primal forward problem* (23)* and* $G_i, G_j$ *the corresponding solutions to the Galerkin dual forward problems* (24).* Then,*

27 $$\begin{aligned} Q(u_i(\sigma ) - u_j(\sigma )) = b(u_i(\sigma ) - u_j(\sigma ), G_i(\sigma ) - G_j(\sigma )). \end{aligned}$$

#### *Proof*

Taking into account (24), (26) and $\overline{X}_j \subset X_i \subset H_0^1(\varOmega )$, we have that

$$\begin{aligned} Q(u_i(\sigma) - {u_j}(\sigma ))
&= b(\sigma; {u_i}(\sigma ) - {u_j}(\sigma), {G_i}(\sigma))\\
&= b(\sigma; u_i(\sigma), {G_i}(\sigma)) - b(\sigma; u_j(\sigma), G_i(\sigma )) \\
&= b(\sigma ; u_i(\sigma ), G_i(\sigma )) - b(\sigma; u_j(\sigma), G_i(\sigma )) \\
& \quad - F(G_j(\sigma )) + b(\sigma ; u_j(\sigma),G_j(\sigma))\\
&= b(\sigma ; u_i(\sigma ), G_i(\sigma )) - b(\sigma; u_i(\sigma),G_j(\sigma ))\\
& \quad - b(\sigma ; u_j(\sigma ), G_i(\sigma )) + b(\sigma; u_j(\sigma ), G_j(\sigma )) \\
&= b(\sigma ; u_i(\sigma ), G_i(\sigma ) - G_j(\sigma )) \\
& \quad - b(\sigma ; u_j(\sigma ), G_i(\sigma ) - G_j(\sigma ))\\
&= b(\sigma ; u_i(\sigma ) - u_j(\sigma ), G_i(\sigma)-G_j(\sigma)). \end{aligned}$$

$$\square $$

### Logging curve

Taking into account $N$ positions of the probe and denoting by $q^i$ the intensity of current sources imposed by their position, we obtain a vector of primal forward problems:

28 $$\begin{aligned} \left\{ \begin{array}{l} \text{ Find }u^i(\sigma ) \in H_0^1(\varOmega ) \hbox { such that:} \\ \displaystyle \;b(\sigma ; u^i(\sigma ), v) = F^i(v) \; \; \forall v \in H_0^1(\varOmega ), \end{array}\right. \end{aligned}$$

where

29 $$\begin{aligned} F^i: H_0^1(\varOmega ) \ni v \rightarrow (q^i, v)_{L^2(\varOmega )} \in {\mathbb {R}}. \end{aligned}$$

 Let us define next the vector of the influence operators $Q^i \in H^{-1}(\varOmega )$, so that

30 $$\begin{aligned} H_0^1(\varOmega ) \ni v \rightarrow \; < Q^i, v > \; = \frac{1}{\text{ meas }(\varOmega _P^i)}\int \limits _{\varOmega _P^i} v \, dx \in {\mathbb {R}}, \end{aligned}$$

for $i = 1, \dots , N$ and $\varOmega _P^i \subset \varOmega$ being the domains occupied by the probe’s receiver at its consecutive positions. Now, we will define a vector of dual problems:

31 $$\left\{ \begin{array}{l}\text{Find} G^i(\sigma ) \in H_0^1(\varOmega ) \hbox { such that}: \\ \displaystyle b(\sigma ; w, G^i(\sigma )) = Q^i(w) \; \; \forall w \in H_0^1(\varOmega ). \end{array}\right. $$

Remark 3 and (29) imply immediately that

32 $$\begin{aligned} ( q^i, G^i(\sigma ) )_{L^2(\varOmega )} = F^i(G^i(\sigma )) = Q^i(u^i(\sigma )), \end{aligned}$$

where $u^i(\sigma ), G^i(\sigma )$ are the solutions of the primal and dual problems (28) and (31), respectively.

**Fig. 2:**
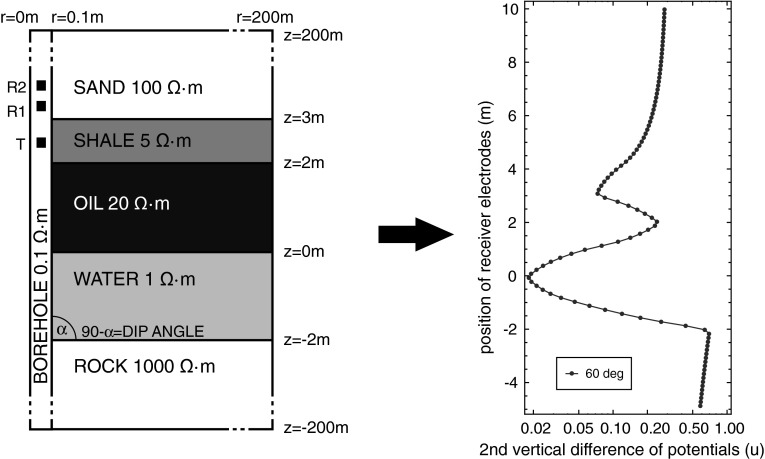
The computation of the sample *logging curve* consists of solving a sequel of multiple forward problems (28) over a domain composed of a *borehole* and five formation layers with assumed conductivities. This set of layers will be utilized in the experimental section

The vector $Q^i(u^i(\sigma )), \; i=1,\ldots ,N$ being the ordered collection of values of the indicator functionals obtained for the consecutive positions of the probe, will be called a *logging curve*. Its coordinates might be expressed by the dual solution or by both primal and dual solutions (see (32)). In other words, computing the logging curve will consist of solving a sequel of forward dual problems (28) respecting the assumed resistivities (see e.g. Fig. 2).

#### *Remark 4*

Notice that all features proved for the primal forward and dual problems (as existence and uniqueness of solution, continuous dependency on right-hand sides as well as convergence of Galerkin approximations) are true for each of the logging curve component problem (28).

## Adaptive *hp* finite element strategy for solving forward problems

For the forward simulations, we employ a Finite Element Method (*hp*–FEM) with variable element size *hp* and polynomial order of approximation *hp* throughout the computational grid. Once the problem is solved in a given discretization (mesh), the error associated to the discrete solution is estimated using a reference solution associated to a finer grid. If that error is above a given threshold level, the discretization is enriched either by dividing some elements containing most of the error or by increasing the polynomial order of approximation in certain areas of the domain. After performing these refinements, the quality of the solution is again evaluated using a reference solution in a finer grid, and the entire enrichment procedure is repeated until the final solution exhibits a given degree of accuracy.

To estimate the error of a given *hp*–grid, we employ as a reference solution the one associated to the globally *hp*–refined grid, *i.e.*, the $h/2,p+1$-mesh. Details on the automatic refinement strategy can be found in Demkowicz (2006), Demkowicz et al. (2007).

The main advantage of the self-adaptive *hp*–FEM is that it delivers exponential convergence rates in terms of the error vs. the number of unknowns for the problems considered in this paper (elliptic problems with a piecewise analytic solution). A proof of this result can be found in Babuška and Guo (1996) and references therein (including Gui and Babuška 1986a, b). Notice that other versions of the FEM (including $h$– and *hp*–FEM) converge at best algebraically.

In order for the error to converge exponentially fast in a particular quantity of interest (solution at receivers) rather than in a global energy norm, we employ a modification of the traditional energy-norm based *hp*–adaptive strategy called goal-oriented *hp*–adaptive strategy.

Such refinement strategy employs the solution of a dual (adjoint) problem (15) to estimate the error in the quantity of interest (see Pardo et al. (2006b) for details).

Let us denote the relative error of the primary forward problem (12) by $e_{rel}$ and by $\epsilon _{rel}$ the relative error of the dual forward problem being the difference between two consecutive approximate solutions obtained by the goal-oriented *hp*–FEM.

In particular, the exponential convergence of the self-adaptive goal oriented *hp*–FEM is experimentally confirmed as the straight line $y=-ax+b$ in the system of coordinates, where horizontal axis represents the cube root of the number of degrees of freedom $x=N^{1/3}$ and vertical axis represents the logarithm of the relative error $y=log_{10}(\left\| e_{rel} \right\| ), \; \left\| e_{rel} \right\| < 1$, where $\left\| \cdot \right\|$ denotes the proper norm in the space of forward primal problem solutions. The constants $a$ and $b$ are positive $a,b>0$ and problem dependent. This implies the following relation

33 $$\begin{aligned} log_{10}(\left\| e_{rel} \right\| )=-a(N^{1/3}) + b, \; \left\| e_{rel} \right\| < 1, \end{aligned}$$

which in turn implies

34 $$\begin{aligned} N = -c_1 (log_{10}(c_2 \left\| e_{rel} \right\| ))^3, \; \left\| e_{rel} \right\| < 1, \end{aligned}$$

where the constants are problem specific $c_1=a^{-3}, c_2=10^{-b} >0$. The computational cost of the solution of the problem by using direct solver over the two dimensional mesh, depends on the structure of the *hp* refined mesh. For regular mesh the cost is of the order $O\left( N^{3/2}\right)$. For meshes with point-wise singularities the cost can be reduced down to the linear one $O\left( N\right)$. Finally,

35 $$\begin{aligned} cost = O \left( - c_1 (log_{10}(c_2 \left\| e_{rel} \right\| ))^{3r} \right) , \; \left\| e_{rel} \right\| < 1, \end{aligned}$$

where $r\in [1,3/2]$, and this time $c_1=a^{-3r}, c_2=10^{-b} >0$.

Having *hp*–FEM primary forward solution, the dual forward solution can be obtained with a linear computational cost $O(N)$. We can utilize the LU factorization of the primal problem matrix, and perform one additional forward and backward substitution. Thus, the relation between the computational cost and relative error has the same form (35) for primal and dual problems solution (they may differ only in value of constant $c_1$).

## Inverse problem

We intend to find the best approximation of the unknown resistivities (inverse of the conductivity field) $\rho = \frac{1}{\sigma }$ having measured the logging curve $Q^i(u^i(\sigma )), \; i=1,\ldots ,N$.

Let us define the search domain as

36 $$ \begin{aligned} \begin{array}{lll} {\mathcal {D}} & = & \big\{ \omega \in L^\infty (\varOmega ); \; \exists M, c_0; M > c_0 > 0;\\ &{} &{} \frac{1}{M} \le \omega \le \frac{1}{c_0}, \text{ almost } \text{ everywhere } \text{ in } \; \varOmega \big\}. \end{array} \end{aligned} $$

The dual inverse problem may be defined as follows:

Find $\hat{\omega } \in {\mathcal {D}}$ such that:

37 $$\begin{aligned} \begin{array}{l} \lim\limits _{h\rightarrow 0,\, p\rightarrow + \infty } \left\{ \sum \limits _{i=1}^N \left| F^i\left (G^i_{h,\,p}\left(\frac{1}{\hat{\omega }}\right )\right ) - F^i\left (G^i\left(\frac{1}{\rho }\right)\right ) \right| \right\} \\ \le \\ \lim\limits _{h\rightarrow 0,\, p\rightarrow + \infty } \left\{ \sum \limits _{i=1}^N \left| F^i\left (G^i_{h,\,p}\left (\frac{1}{\omega }\right )\right ) - F^i\left (G^i\left (\frac{1}{\rho }\right )\right ) \right| \right\} \\ \quad\quad\;\forall \omega \in {\mathcal {D}}, \end{array} \end{aligned}$$

where $\rho = \frac{1}{\sigma } \in {\mathcal {D}}$ denotes exact parameters, $\omega$ denotes approximated parameters, $G^i\big (\frac{1}{\rho }\big )$ is the exact solution to the dual problem (31) for the $i$-th position of the probe associated with the $i$-th point of the logging curve for the exact parameters $\rho$, and $G_{h,p}^i\big (\frac{1}{\omega }\big )$ is the approximate (by $hp$–FEM) solution to the dual problem (31) for the $i$-th position of the probe associated with the $i$-th point of the logging curve for the approximated parameter $\omega$. Moreover $F^i \in H^{-1}(\varOmega )$ is such that $F^i(v) = (q^i, v)_{L^2(\varOmega )} \; \forall v \in H^1_0(\varOmega )$.

Taking into account (19) in Remark 3, we can rewrite the above problem (37) to the equivalent form:

Find $\hat{\omega } \in {\mathcal {D}}$ such that:

38 $$\begin{aligned} \begin{array}{l} \lim\limits _{h\rightarrow 0, p\rightarrow + \infty } \left\{ \sum \limits _{i=1}^N \left| Q^i\Big (u^i_{h,\,p}\big (\frac{1}{\hat{\omega }}\big )\Big ) - Q^i\Big (u^i\big (\frac{1}{\rho }\big )\Big ) \right| \right\} \\ \le \\ \lim\limits _{h\rightarrow 0, p\rightarrow + \infty } \left\{ \sum \limits _{i=1}^N \left| Q^i\Big (u^i_{h,\,p}\big (\frac{1}{\omega }\big )\Big ) - Q^i\Big (u^i\big (\frac{1}{\rho }\big )\Big ) \right| \right\}  \forall \omega \in {\mathcal {D}}. \end{array} \end{aligned}$$

**Fig. 3:**
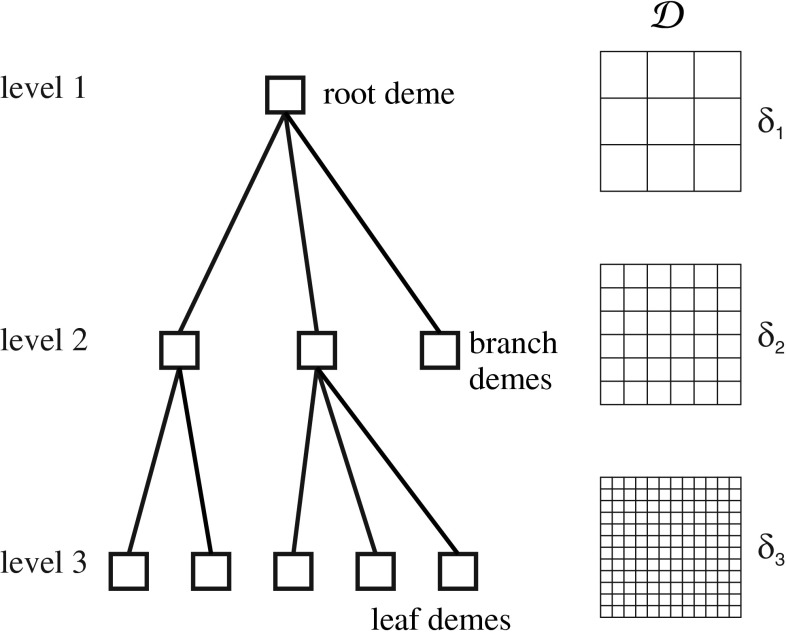
HGS tree and corresponding coding meshes for binary implementation

In other words, for a given reference logging curve, geometry of the formation layers and resistivities of the borehole and top and bottom formations, we seek for $\hat{\omega }$ resistivities of the formation layers. The reference logging curve is usually obtained from the field measurements. The idea of the inverse logging curve problem was illustrated by the simple example of finding 3 parameters $(\omega _0, \omega _1, \omega _2)$ being the constant value of the resistivity function $\hat{\omega }$ (see Fig. 3).

## Hierarchic genetic strategy for solving global optimization problems

**Fig. 4:**
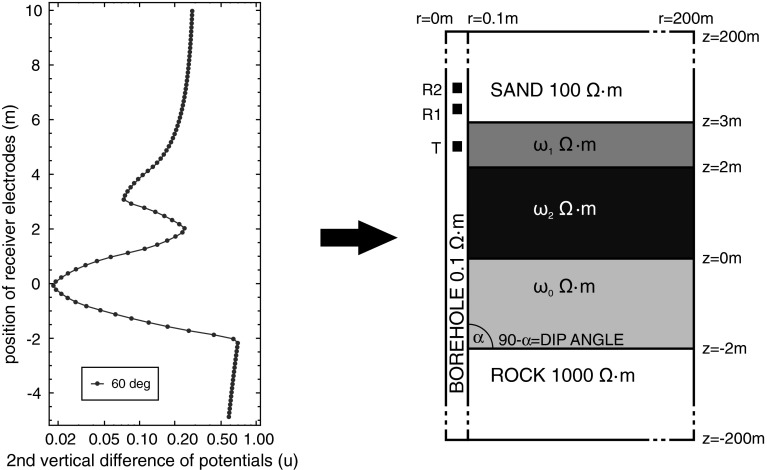
The inverse problem is to find resistivities of formation layers from a given *logging curve* (for details of this example refer to Sect. 9)

The hierarchic genetic strategy (HGS) produces a tree-structured set of concurrent evolutionary processes (see Fig. 4). HGS was introduced in Schaefer and Kołodziej (2003). The structure of the tree changes dynamically and its depth is bounded by $m < + \infty$.

HGS performs calculations in the following way:

- The first deme (population) of order one is created. There is always exactly one deme at the first level and it is called the *root deme*. The root-deme performs a chaotic search with low accuracy.
- After a fixed number of genetic epochs $K$ called the *metaepoch*, each parental deme (at level $< m$) selects its best fitted individual and *sprouts* a child-deme in the neighborhood of this individual. Sprouting new demes is repeated concurrently for root and all active demes laying below in the HGS tree (at levels $< m$), called *branches*, excluding deepest demes (at level $m$), called *leaves*.
- Demes at the consecutive levels search with higher and higher accuracy. The maximum, target accuracy in the global phase is performed by leaves.
- To prevent redundancy, HGS implements *conditional sprouting* and *branch reduction*. The former allows new demes to be sprouted only in regions, which are not explored by demes already activated at the particular level of the HGS tree. The latter reduces (joins and jointly selects) demes at the same level that perform search in the common landscape region or in the regions that were already explored.

The HGS stopping policy is composed of a local branch stopping conditions that terminates the evolution in leaves and branches, and a global stopping condition that evaluates the total maturity of the global search. Local stopping conditions monitor progress of evolution in deme and stop it, if unsatisfactory. The whole strategy might be stopped if no new demes are sprouted after a sufficiently large number of metaepochs and all active leaves were stopped. The other possibility is to stop the strategy when the satisfactory number of well fitted individuals were already found. Some details of stopping policy for logging measurements inversion will be explained later in Sect. 7.

The strategy was implemented and studied twofold: using binary encoding and SGA engines (see Kołodziej et al. 2004a; Schaefer and Kołodziej 2003) for each branch and leaf, and using real-number encoding and Simple Evolutionary Algorithms (SEA) for running evolution of each deme (see Wierzba et al. 2003).

In the binary version of HGS, we use various encoding precisions and changing length of binary genotypes in demes at different levels, to obtain different search accuracies. The length of a genotype increases along with increasing level in the HGS tree. We apply a hierarchical nested encoding to obtain search coherency for populations at different levels: we begin with defining the densest mesh of phenotypes in ${\mathcal {D}}$ for populations at $m$-th level and recursively select some nodes to create meshes for lower-order demes (see Fig. 4). The maximum diameter of the mesh $\delta _j$ (satisfying $\delta _m < \ldots < \delta _1$) determines the search accuracy at $j$-th level of the HGS tree.

In the real-number encoding version of HGS, a genotype is a vector of floating point numbers. In order to introduce a sequence of increasing genetic spaces for subsequent orders of branches, we use a sequence of scaling coefficients $+\infty > \eta _1 \ge \eta _2 \ge \ldots \ge \eta _m = 1$. Let us denote a search domain by ${\mathcal {D}} = \prod _{i=1}^N [a_i, b_i] \subset {\mathbb {R}}^N$, where $a_i, b_i; \; a_i < b_i$ are the lower and upper bounds for $i$-th decision variable. The genetic space at $i$-th level is defined as $\prod _{i=1}^N [0,\frac{b_i - a_i}{\eta _i}] \subset {\mathbb {R}}^N$. In this way, we obtain genetic spaces that are smaller for lower level branches, closer to the root. The genetic space for leaves $\prod _{i=1}^N [0,(b_i - a_i)]$ is of the same size as the admissible domain ${\mathcal {D}}$, and has the richest numerical representation. If a target search accuracy in leaves equals $\delta _m$, the accuracy in the underlying demes will be reduced to $\delta _j = \eta _j \, \delta _m$, for $j = 1, \ldots , m-1$.

Asymptotic analysis of HGS with binary encoding was studied in Schaefer and Kołodziej (2003). It was proved that the strategy possesses an asymptotic guarantee of success. The decrease of computational cost vs. the single population SGA with the finest encoding, represented in HGS leaves was also estimated. HGS application to other inverse problems was shown in Kołodziej et al. (2004a, b). Real-number HGS, along with its efficiency, is discussed in Wierzba et al. (2003).

## Relation between approximate forward and inverse solutions errors

### Estimation for a single position of the probe

Let us denote by $e_{rel}\big (\frac{1}{\omega }\big ) = u_{h/2,p+1}\big (\frac{1}{\omega }\big ) - u_{h,p}\big (\frac{1}{\omega }\big )$ the relative error of the $hp$–FEM solution to the primal forward problem (28) and, similarly, denote by $\epsilon _{rel}\big (\frac{1}{\omega }\big ) = G_{h/2,p+1}\big (\frac{1}{\omega }\big ) - G_{h,p}\big (\frac{1}{\omega }\big )$ to the relative error of the $hp$–FEM solution of the dual forward problem (15) for some $\omega \in {\mathcal {D}}$.

Let us compute now:

$$\begin{aligned} Q\left( u_{h/2,p+1}\left( \frac{1}{\omega }\right) \right) - Q\left( u\left( \frac{1}{\rho }\right) \right)&= Q\left( u_{h/2,p+1}\left( \frac{1}{\omega }\right) - u_{h,p}\left( \frac{1}{\omega }\right) \right) \\&\quad + \, Q\left( u_{h,p}\left( \frac{1}{\omega }\right) - u\left( \frac{1}{\omega }\right) \right) + Q\left( u\left( \frac{1}{\omega }\right) - u\left( \frac{1}{\rho }\right) \right) \\&= Q\left( e_{rel} \left( \frac{1}{\omega }\right) \right) + Q\left( u_{h,p}\left( \frac{1}{\omega }\right) - u\left( \frac{1}{\omega }\right) \right) \\&\quad + \, Q\left( u\left( \frac{1}{\omega }\right) - u\left( \frac{1}{\rho }\right) \right) . \end{aligned}$$

Using Lemma 2 for $e_{rel}\left( \frac{1}{\omega }\right)$ and $\epsilon _{rel}\left( \frac{1}{\omega }\right)$, we have $|Q\left( e_{rel}\left( \frac{1}{\omega }\right) \right) | = |b\left( e_{rel}\left( \frac{1}{\omega }\right) , e_{rel}\left( \frac{1}{\omega }\right) \right) |$ and

39 $$\begin{aligned} \left| Q\left( u_{h/2,p+1}\left( \frac{1}{\omega }\right) \right) - Q\left( u\left( \frac{1}{\rho }\right) \right) \right|&\le L \; \left\| e_{rel}\left( \frac{1}{\omega }\right) \right\| _{H^1_0(\varOmega )} \, \left\| \epsilon _{rel}\left( \frac{1}{\omega }\right) \right\| _{H^1_0(\varOmega )}\nonumber \\&\quad + \, \left\| Q \right\| _{H^{-1}(\varOmega )} \; \left\| u_{h,p}\left( \frac{1}{\omega }\right) - u\left( \frac{1}{\omega }\right) \right\| _{H^1_0(\varOmega )}\nonumber \\&\quad + \, \left\| Q \right\| _{H^{-1}(\varOmega )} \; \left\| u\left( \frac{1}{\omega }\right) - u\left( \frac{1}{\rho }\right) \right\| _{H^1_0(\varOmega )},\end{aligned}$$

*where* $L > 0$ is the continuity constant of the bilinear form $B$.

#### **Proposition 1**

*Taking into account the assumptions of Lemma 1, it is easy to prove that*

40 $$\begin{aligned} \begin{array}{c} \left\| u\left( \frac{1}{\omega ^1}\right) - u\left( \frac{1}{\omega ^2}\right) \right\| _{H^1_0(\varOmega )} \le C \, \left\| \omega ^1 - \omega ^2 \right\| _{L^\infty (\varOmega )}, \end{array} \end{aligned}$$

*where* $\omega ^i = \frac{1}{\sigma ^i}, \; i=1,2$ *and now* $C = \frac{1}{(c_0)^4} \left\| F \right\| _{H^{-1}(\varOmega )}$.

Using (40) and (39) we are able to formulate the target evaluation for the single position of the probe:

#### **Proposition 2**

*The absolute indicator functional error by solving the* $hp$–*FEM dual inverse problem is evaluated by the product of relative* $hp$–*FEM errors of primal and dual solutions added to the absolute* $hp$–*FEM error of primal solution and the accuracy of solving the inverse problem i.e.*

41 $$\begin{aligned} \left| Q\left( u_{h/2,p+1}\left( \frac{1}{\omega }\right) \right) - Q\left( u\left( \frac{1}{\rho }\right) \right) \right|&\le L \; \left\| e_{rel}\left( \frac{1}{\omega }\right) \right\| _{H^1_0(\varOmega )} \, \left\| \epsilon _{rel}\left( \frac{1}{\omega }\right) \right\| _{H^1_0(\varOmega )}\nonumber \\&\quad + \, \left\| Q \right\| _{H^{-1}(\varOmega )} \; \left\| u_{h,p}\left( \frac{1}{\omega }\right) - u\left( \frac{1}{\omega }\right) \right\| _{H^1_0(\varOmega )}\nonumber \\&\quad + \, \left( \frac{1}{(c_0)^4} \left\| Q \right\| _{H^{-1}(\varOmega )} \; \left\| F \right\| _{H^{-1}(\varOmega )} \right) \left\| \omega - \rho \right\| _{L^\infty (\varOmega )}, \end{aligned}$$

*where c*_0_ *and L stand for the coercivity and Lipschitz continuity constants of* $B$,* respectively*.

### Estimation for the dual inverse problem

The estimation delivered by (41) in Proposition 2 will be true for each pair of component problems (28), (31) for $i = 1, \ldots , N$

42 $$\begin{aligned} \left| Q^i\left( u^i_{h/2,p+1}\left( \frac{1}{\omega }\right) \right) - Q^i\left( u\left( \frac{1}{\rho }\right) \right) \right|&\le L \; \left\| e^i_{rel}\left( \frac{1}{\omega }\right) \right\| _{H^1_0(\varOmega )} \, \left\| \epsilon ^i_{rel}\left( \frac{1}{\omega }\right) \right\| _{H^1_0(\varOmega )}\nonumber \\&\quad + \, \left\| Q^i \right\| _{H^{-1}(\varOmega )} \; \left\| u^i_{h,p}\left( \frac{1}{\omega }\right) - u^i\left( \frac{1}{\omega }\right) \right\| _{H^1_0(\varOmega )}\nonumber \\&\quad + \, \left( \frac{1}{(c_0)^4} \left\| Q^i \right\| _{H^{-1}(\varOmega )} \; \left\| F^i \right\| _{H^{-1}(\varOmega )} \right) \left\| \omega - \rho \right\| _{L^\infty (\varOmega )},\nonumber \\ \end{aligned}$$

where $e^i_{rel}\big (\frac{1}{\omega }\big ) = u^i_{h/2,p+1}\big (\frac{1}{\omega }\big ) - u^i_{h,p}\big (\frac{1}{\omega }\big )$ and $\epsilon _{rel}\big (\frac{1}{\omega }\big ) = G^i_{h/2,p+1}\big (\frac{1}{\omega }\big ) - G^i_{h,p}\big (\frac{1}{\omega }\big )$. Summing both sides of the above inequality we obtain

43 $$\begin{aligned} \sum _{i=1}^N \left| Q^i\left( u_{h/2,p+1}\left( \frac{1}{\omega }\right) \right) - Q^i\left( u\left( \frac{1}{\rho }\right) \right) \right|&\le \; L \; \sum _{i=1}^N \left\| e^i_{rel}\left( \frac{1}{\omega }\right) \right\| _{H^1_0(\varOmega )} \, \left\| \epsilon ^i_{rel}\left( \frac{1}{\omega }\right) \right\| _{H^1_0(\varOmega )}\nonumber \\&\quad + \, \sum _{i=1}^N \left\| Q^i \right\| _{H^{-1}(\varOmega )} \; \left\| u^i_{h,p}\left( \frac{1}{\omega }\right) - u^i\left( \frac{1}{\omega }\right) \right\| _{H^1_0(\varOmega )}\nonumber \\&\quad + \, \frac{1}{(c_0)^2} \left( \sum _{i=1}^N \left\| Q^i \right\| _{H^{-1}(\varOmega )} \; \left\| F^i \right\| _{H^{-1}(\varOmega )} \right) \left\| \omega - \rho \right\| _{L^\infty (\varOmega )}.\nonumber \\ \end{aligned}$$

The first component of the right-hand side might be evaluated using Cauchy-Schwarz inequality

44 $$\begin{aligned}&L \; \sum _{i=1}^N \left\| e^i_{rel}\left( \frac{1}{\omega }\right) \right\| _{H^1_0(\varOmega )} \, \left\| \epsilon ^i_{rel}\left( \frac{1}{\omega }\right) \right\| _{H^1_0(\varOmega )}\nonumber \\&\quad \le \; L \, C \; \left( \sum _{i=1}^N \left\| e^i_{rel}\left( \frac{1}{\omega }\right) \right\| _{H^1_0(\varOmega )} \right) \left( \sum _{i=1}^N \left\| \epsilon ^i_{rel}\left( \frac{1}{\omega }\right) \right\| _{H^1_0(\varOmega )} \right) , \end{aligned}$$

where $C$ is the norm equivalence constant in ${\mathbb {R}}^N$. We are ready to formulate the final estimation.

#### **Proposition 3**

*The norm of the logging curve error might be evaluated as the product of norms of relative* $hp$–*FEM errors of primal and dual solutions added to the norm of absolute* $hp$–*FEM errors of primal solutions obtained for all coordinates of the logging curve and the accuracy of solving the inverse problem i.e.*

45 $$\begin{aligned} \sum _{i=1}^N \left| Q^i\left( u^i_{h/2,p+1}\left( \frac{1}{\omega }\right) \right) - Q^i\left( u\left( \frac{1}{\rho }\right) \right) \right|= \sum _{i=1}^N \left| F^i\left( G^i_{h/2,p+1}\left( \frac{1}{\omega }\right) - G^i\left( \frac{1}{\rho }\right) \right) \right|\nonumber \\\,\le \; C' \left( \sum _{i=1}^N \left\| e^i_{rel}\left( \frac{1}{\omega }\right) \right\| _{H^1_0(\varOmega )} \right) \nonumber \\\quad \left( \sum _{i=1}^N \left\| \epsilon ^i_{rel}\left( \frac{1}{\omega }\right) \right\| _{H^1_0(\varOmega )} \right) \nonumber \\ \quad\quad\quad+ C'' \,\sum _{i=1}^N \; \left\| u^i_{h,p}\left( \frac{1}{\omega }\right) - u^i\left( \frac{1}{\omega }\right) \right\| _{H^1_0(\varOmega )}\nonumber\\+C'''  \left\| \omega - \rho \right\| _{L^\infty (\varOmega )}. \end{aligned}$$

*where*

$$\begin{aligned} \begin{array}{l} C' = L \, C,\\ C'' = \max\limits _{i=1,\ldots ,N} \left\{ \left\| Q^i \right\| _{H^{-1}(\varOmega )} \right\} ,\\ C''' = \frac{1}{(c_0)^4} \left( \sum \limits _{i=1}^N \left\| Q^i \right\| _{H^{-1}(\varOmega )} \; \left\| F^i \right\| _{H^{-1}(\varOmega )} \right) \end{array} \end{aligned}$$

*and* $c_0, \, L$ *stand for the coercivity and Lipschitz continuity constants of* $B$,* respectively, and* $C$ *is the Cauchy-Schwarz constant in* ${\mathbb {R}}^N$.

Let us apply the above Proposition 3 to the fitness evaluation at the $j$-th level of the $hp$–HGS tree.

#### *Remark 5*

The first right-hand side component of (45) expresses the influence of the limit relative direct errors (primal and dual ones) imposed on the $hp$–FEM refinement process. The second one is proportional to the absolute FEM error, which decreases to 0 during $hp$ refinements (see Remark 4). The third component is evaluated from below by $C''' \delta _i$, where $\delta _j$ expresses the error appearing in the inverse search performed by the $j$-th level HGS branch (the grid size in case of binary implementation). In order to make the $hp$–HGS inversion on the $j$-th level computationally economic, we should keep the first and the third component comparable. In different words, decreasing $\left\| e^i_{rel}(\frac{1}{\omega }) \right\| _{H^1_0(\varOmega )}$ and $\left\| \epsilon ^i_{rel}(\frac{1}{\omega }) \right\| _{H^1_0(\varOmega )}$ below the quantity $Ratio(j)=\frac{1}{N}\sqrt{\delta _j\frac{C'''}{C'}}$ does not improve the accuracy of fitness evaluation.

## The adaptive strategy for solving dual inverse problems

The mathematical results concerning relations between the approximate forward and inverse errors (Proposition 3 and Remark 5) allow to apply the HGS strategy for inversion of 3D DC resistivity logging measurements (38) in an exceptionally economic way.

The misfit evaluation needs to solve the series of forward problems (15) associated with each point of the logging curve. Computational cost of solving the forward problem by the $hp$–FEM (see formula (34) in Sect. 3) depends strongly on the assumed accuracy. We are able to apply cheap, low accuracy misfit evaluations during evolution in root-deme. The accuracy of misfit evaluation will grow deep into the HGS tree, according to the ratio introduced by Remark 5, up to the maximum one in leaves. The resulted strategy is called $hp$–HGS.

A typical configuration of $hp$–HGS tree imposes large root-deme and strongly decreasing size of branch-demes up to the smallest one for a leave-demes containing only several individuals.

Both, conditional sprouting and branch reduction mechanisms (see Sect. 5) are based on a distance analysis performed in the phenotype space. In the first case, the distance between the seed individual (the best fitted individual distinguished from the parental deme and re-coded to the consecutive, child-level of the $hp$–HGS tree) and the centroids of demes already sprouted at the child-level is tested. If this distance is lower then the assumed threshold, sprouting operation is abandoned. The threshold is frequently set as the double of mutations standard deviation at the child-deme level. In the current $hp$–HGS version, we restricted the range of distance comparison to the child-demes of the sprouting parental one.

Similarly, branch reduction is based on a distance between centroids of two demes at the same level of the $hp$–HGS tree. If it is smaller than the threshold (usually set as a mutation’s standard deviation at the particular level of the $hp$–HGS tree), the union of both demes is commonly selected, creating a new deme, whose evolution is continued. Branch reduction mechanism is invoked periodically, after each assumed number of metaepochs.

Local stopping conditions monitor the progress of a mean fitness in branches and leaves. If it does not decrease more than an assumed value in the prescribed number of epochs, the evolution of this deme is abandoned. The stopping parameters are set to be restrictive, i.e. they usually allow to make only several most effective steps of evolution. Generally, it is more economic to sprout new demes than to run ineffective ones for a long time.

The whole $hp$–HGS is stopped when a satisfactory number of well fitted individuals is found. It is possible to define a satisfactory fitted individual in the case of inverse problems, because its minimum value (the minimum misfit value) is always zero. The number and possible location of minimizers might be assessed by experts on the field of petroleum and gas survey.

A brief description of the $hp$–HGS strategy is presented in the form of a pseudocode (Algorithms 1, 2). 
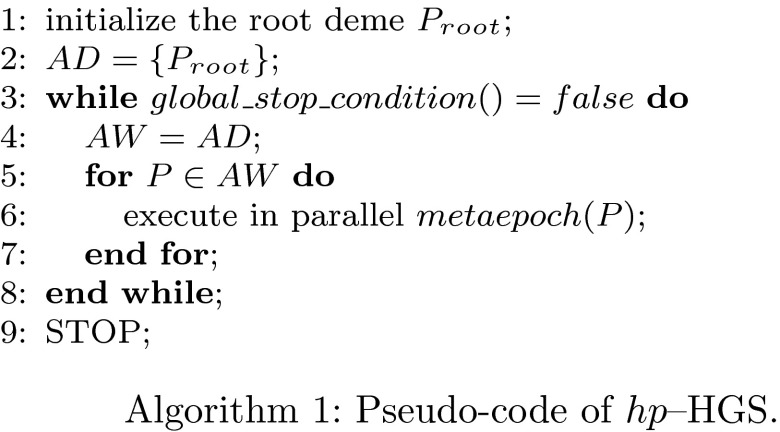


In the first algorithm, we use sets $AD$ and $AW$ to store alive demes. The function $global\_stop\_condition()$ checks if either a satisfactory solution has been found or no more local extrema can be found. 
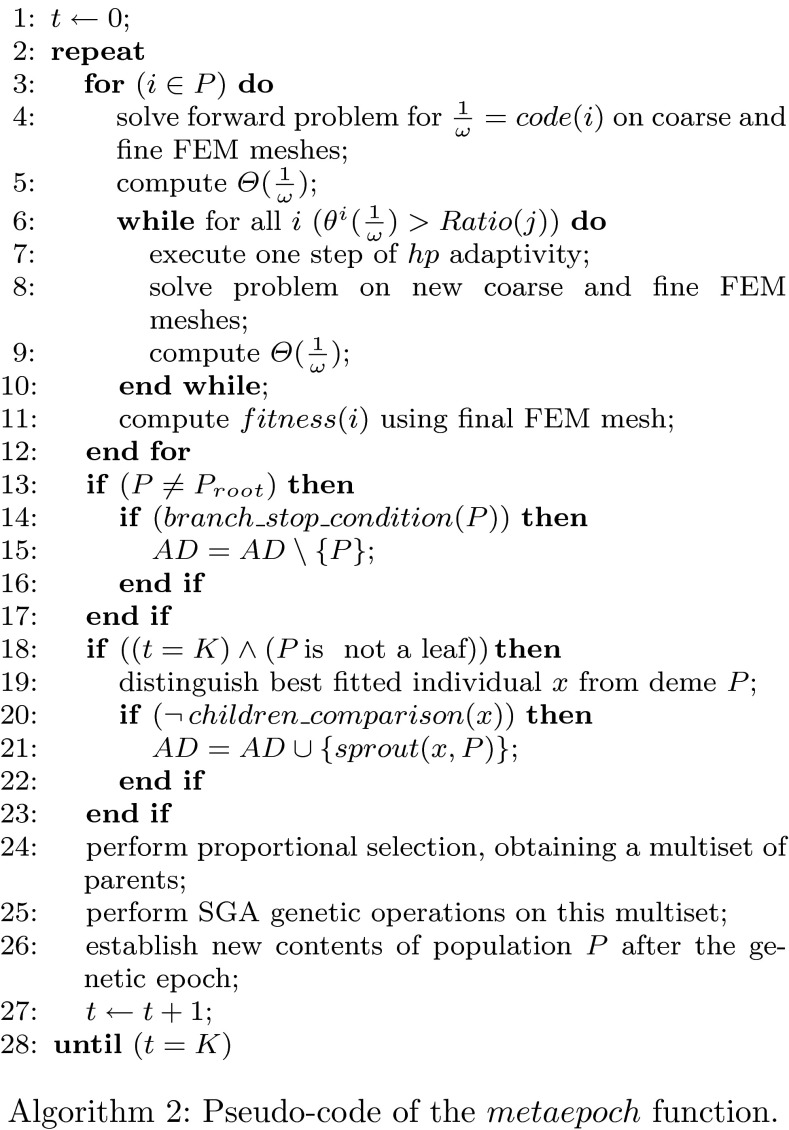


Let us denote by $\Theta (\frac{1}{\omega }) = (\theta ^1(\frac{1}{\omega }), \ldots , \theta ^N(\frac{1}{\omega }))$, where $\theta ^i(\frac{1}{\omega }) = \left\| \epsilon ^i_{rel}(\frac{1}{\omega }) \right\| _{H^1_0(\varOmega )}$ the vector of relative errors of $hp$–FEM appearing by the solving direct problems (15) for all logging curve points $i = 1,\ldots ,N$. The $hp$-adaptation of the FEM solution of the forward dual problem is performed until at least one quantity $\theta ^i(\frac{1}{\omega })$ is below than or equal to the assumed $Ratio(j)$ (see Remark 5).

The function $branch\_stop\_condition(P)$ returns $true$ if it detects a lack of evolution progress of the current deme $P$. The generic function $fitness(i)$ computes fitness accordingly to the position of $P$ in the $hp$–HGS tree.

The *conditional sprouting* mechanism is implemented as follows. The procedure $children\_comparison(x)$ compares the phenotype averages (centroids) of all child-demes with the phenotype of the best fitted individual $x$ distinguished from the parental deme $P$. This procedure returns $true$ if $x$ is sufficiently close to the centroids of the existing child-demes. The generic function $sprout(x,P)$ returns a new child-deme surrounding $x$ using proper encoding and sampling, according to the position of the parental deme $P$ in the $hp$–HGS tree.

Lines 15 and 21 in Algorithm 2 are mutually excluded among all instances of $Metaepoch(P)$ function processing in parallel, because the set of active demes $AD$ constitutes a common, shared data. A particular implementation-based mechanism of critical section handling is applied. The modifications of the set of alive demes $AD$, imposed by the particular deme $P$ (see lines 15 and 21 in the $Metaepoch$ routine), do not influence changes performed by other demes, because of their tree structure (see Fig. 4). The *branch reduction* mechanism is not described in the Algorithms 1 and 2 for the sake of simplicity.

The presented general algorithmic description constitutes a basis for the various implementations. The serial (trivial) one forces to execute the loop 5–7 in Algorithm 1 sequentially. The highly developed structure of $hp$–HGS demes creates an opportunity for advanced coarse grained distributed implementations. Notice, that the fitness evaluations costs dominate and are several degree of magnitude higher than all other costs associated with individuals and demes handling (mutation, crossover, sprouting, branch reduction, etc.) when solving parametric inverse problems. Thus a typical implementation runs all operation except fitness evaluation on a single computer node (e.g., front-end workstation or single node of a cluster), while the $hp$–FEM solving forward problems are computed in parallel, dynamically scheduled to multiple sub-complexes of computational nodes distinguished from a high performance cluster. Local optimization methods utilized in the second phase are scheduled in a similar way. We refer to Grochowski et al. (2006), Jojczyk and Schaefer (2009), Momot et al. (2004) for the more advanced agent-based scheduling of hierarchic genetic computations in a distributed environment.

## Short discussion of hybrid strategy features

We synthesize the main advantages of the proposed hybrid strategy:

It can find all minima of misfit after a sufficient number of steps, which results from the asymptotic guarantee of success of the global phase, performed by $hp$–HGS (see Schaefer and Barabasz (2008)). Notice that this result is not trivial in the case of complex multi-deme strategy with adaptive search accuracy. We can obtain at least one well fitted individual in basin of attraction of each global minimizer. The asymptotic guarantee of success allows to study ill–posed inverse problems with ambiguous solutions, which are difficult or even impossible to obtain by other methods.

There are three ways of decreasing the computational cost:

- *By minimizing the number of fitness calls in the global phase*. It is obtained mainly by reducing the size and number of child-demes (branches and leaves). More accurate, intensive searches are mainly activated in promising regions. Moreover, local stopping conditions restrict the evolution in child-demes to the several most effective initial epochs, because it is more economic to sprout new demes than to run ineffective ones for a long time. Conditional sprouting and branch reduction mechanisms result in the additional, significant reduction of the number of active branches and leaves, protecting search redundancy.
- *By common scaling of the forward and inverse search accuracy*. The computational cost of misfit evaluation by the $hp$–FEM rapidly decreases if the accuracy is reduced (see formula (35), Sect. 3). The proper scaling of the forward error with respect to the assumed inverse one at each level of the $hp$–HGS tree (see Proposition 5, Algorithm 2) allows for a cheap, exhaustive exploration in root-deme and branches close to the root. The maximum accuracy of the global phase utilized by leaf-demes is also far from the target one, only sufficient for recognizing and separating basins of attraction of different minimizers.
- *By reducing a number of local searches*. The global phase allows to start only one local search per each recognized basin of attraction. Reduction of the number of local processes is crucial because of their huge computational expense caused by many fitness calls necessary for Hessian approximation, and the high computational cost of a single misfit evaluation with the highest target accuracy.

We compare advantages underlined before with features of competing strategies:

- *Genetic algorithm with a single population and multi-deme, island model*. It performs more fitness calls than $hp$–HGS because it does not concentrate the search in promising areas, it does not use a restrictive stopping condition in order to preserve global search and it may concentrate the search in a single basin of attraction “a premature convergence” for a long time. The above proposition may be supported by the tests for continuum optimization benchmarks (Schaefer and Kołodziej 2003; Kołodziej et al. 2004a; Wierzba et al. 2003). Moreover, in contrast to $hp$–HGS, the considered group of genetic strategies performs all fitness evaluations with a uniform high accuracy, which generates an enormous unacceptable computational cost.
- *Local search with multiple restarts* (*departing from random solutions*). This strategy needs to start a large number of expensive local processes, comparable to the size of a root-deme in $hp$–HGS in order to find multiple minimizers, which generates an unacceptable total computational cost, much greater than in the case of two-phase strategies, in which the number of starting points is significantly reduced (see e.g., Törn (1975)).
- *Memetic algorithm, where the local search is used in the main loop of the GA*.
  Local, convex optimization methods incorporated in evolutionary search as a “gradient mutation” can degrade its exploratory power if activated too frequently. Moreover, too many local searches bound the memetic search to the multi-start strategy, thwarting its efficiency. The core idea in memetic strategies is to gain experience to make a further search more economic . This idea is represented in the proposed strategy, being a composition of $hp$–HGS with local, convex methods. This strategy offers the hierarchy of searches with various degrees of locality. All of them are activated by the main, genetic one, performed by the root-deme. Each path in the $hp$–HGS tree represents stochastic processes that explore the selected region of admissible domain more locally and accurately (without losing an asymptotic guarantee of success). The deeper exploration is undertaken conditionally when it is promising (e.g., the well fitted individual is found in the region penetrated by a parental deme). During this procedure, demes introduced in the same basin of attraction bound one to each other and are reduced by the branch reduction mechanism. The most promising paths reach the leaf-level and point out the basins of attraction of separate minimizers. Most local, expensive convex methods are started from the best fitted individuals in such leaves.

## Experiments

The problem under considerations is the inverse DC problem in which we are searching for the values of three ground layer resistivities $\omega _0$, $\omega _1$ and $\omega _2$. The reference values are $\omega _0 = 1 \ \varOmega \cdot \hbox {m}$, $\omega _1 = 5 \ \varOmega \cdot \hbox {m}$ and $\omega _2 = 20 \ \varOmega \cdot \hbox {m}$. We have performed the following series of computations.

- Global search by means of the $hp$–HGS with the binary encoding.
- Local gradient-based BFGS method started from the points found by the binary HGS.
- Global search by means of the $hp$–HGS with the floating-point encoding.
- Local gradient based BFGS method started from the points found by the floating-point HGS.

In all these simulations, the misfit values are computed as the square of the Euclidean distance (hence: without the square root) between an obtained logging curve and the reference logging curve (the “exact” one).

For the search domain we select the cube $[0.1, 10^3]^3$. To provide a more thorough search for the parameter values around $1$ we transformed the original domain with the following mapping

46 $$\begin{aligned} {\mathbb {R}}^3 \ni x \longmapsto \left[ \log _{10} (x_i) + 1 \right] _{i=1,2,3} \in {\mathbb {R}}^3, \end{aligned}$$

which resulted in the cube $[0,6]^3$.

### Global “binary” search

We performed a simulation of the 3D DC borehole resistivity measurements problem using $hp$–HGS method with three levels. Parameters of the simulation are presented in Table 1. Population sizes were selected to balance the time of evaluating a single solution with search capabilities of a population. Code length for a single parameter was $15$ on the first level, $21$ on the second level, and $27$ in the leaves (third level). Parameters setting discussed above is based on our experience in solving ill-posed inverse parametric problems of heat conduction and elasticity Barabasz et al. 2011a, b, 2009; Paszyński et al. 2007.

**Table 1:** Parameters of the simulation in 3D DC case. The last row corresponds to the maximum relative error decrement in a single $hp$–FEM step applied to the solution of a forward problem

	Level 1	Level 2	Level 3
Population size	12	6	4
Code length	45	63	81
Mutation rate	0.1	0.01	0.001
Crossing rate	0.5	0.5	0.5
Relative $hp$–FEM error	0.7	0.1	0.01

The reference logging curve is usually obtained from the field measurements. For testing purposes, we computed this curve for the $60$ degrees deviated well by using a self-adaptive goal oriented $hp$–FEM algorithm with high accuracy ($10^{-5}$). The model problem is composed of: a borehole with resistivity $0.1\varOmega \cdot m$, a sand layer with resistivity $100 \varOmega \cdot m$, a shale layer with resistivity $5 \varOmega \cdot m$, an oil layer with resistivity $20 \varOmega \cdot m$, a water layer with resistivity $1 \varOmega \cdot m$, and a rock layer with resistivity $1000 \varOmega \cdot m$, which makes a total of five layers, as illustrated in Fig. 3.

Fitness value of each candidate solution $\omega$ (resistivity vector) was evaluated as the square of the Euclidean norm of the difference between discrete representations of the reference logging curve calculated with high accuracy and the logging curve computed by the self-adaptive goal-oriented $hp$–FEM algorithm for $\omega$ with accuracy depending on the level in HGS tree. The accuracy (see last row in Table 1) corresponds to the maximum relative error decrement in a single $hp$–FEM step (see e.g., Paszyński et al. (2010)) applied to the solution of a forward problem at a particular HGS level.

**Fig. 5:**
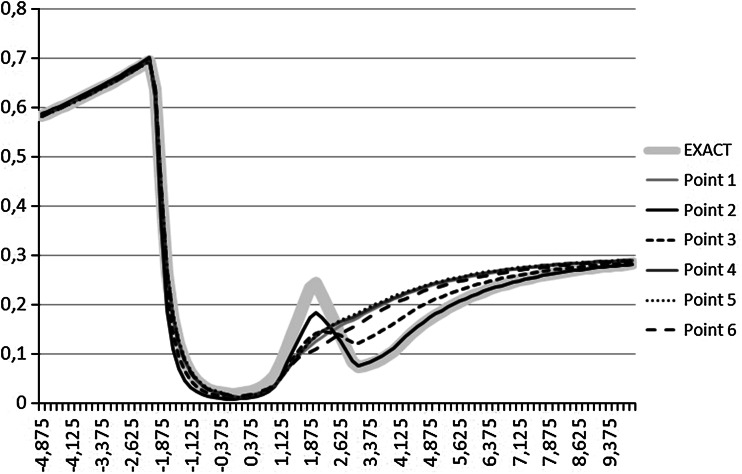
The *logging curves* corresponding to points found after binary global search phase. The *bold gray curve* corresponds to the exact *logging curve*

**Fig. 6:**
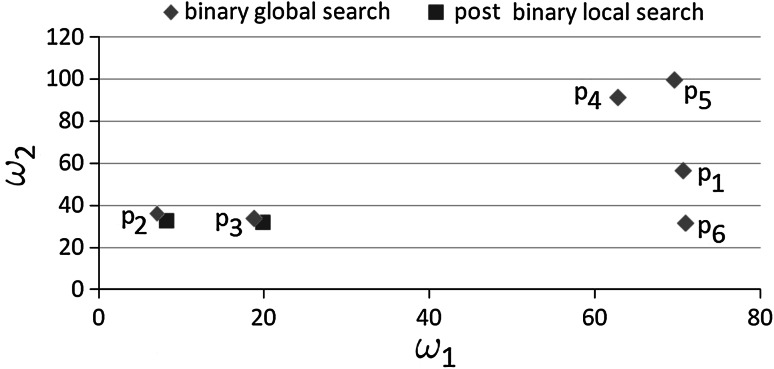
The results of the global and local binary phases. The particular points are denoted by $P_1,....,P_6$ labels, since they correspond to the six curves presented in Fig. 5

The results of the global binary search phase (six obtained points) are presented in Table 2. For testing purposes, we have executed the self-adaptive goal-oriented $hp$–FEM algorithm on these points, in order to generate and plot the resulting logging curves. The curves corresponding to the found six points are presented in Fig. 5. The curves have also been compared to the exact logging curve, denoted by bold light gray color. The best fitted Point 2 is the most similar point to the exact logging curve, as expected. The six points obtained after the global binary phase are also depicted in Fig. 6 by six diamonds. The figure does not present the values of $\omega _0$ since they are all approximately equal to $1$.

**Table 2:** Results of the global binary search

	$\omega _0$	$\omega _1$	$\omega _2$	*Misfit*
Point 1	1.019	70.624	56.998	0.220766696114
Point 2	0.549	6.540	37.186	0.0420046286544
Point 3	0.760	18.789	33.791	0.0919687357984
Point 4	1.022	62.664	91.5683	0.224530498495
Point 5	1.069	69.583	99.774	0.236968057675
Point 6	1.004	70.920	31.900	0.201298418709

### Local “post-binary” search

The local phase was executed from the two best fitted points obtained from the binary global phase. In particular, we have executed the local phase on Point 2 and Point 3, since the misfit of these points is less than $0.1$. We have used the BFGS method, and the relative error of the self-adaptive $hp$–FEM algorithm was set to $0.001$. The local phase has slightly changed the location of the points, as it is depicted in Fig. 6 by squares, located in the neighborhood of Point 2 and Point 3. On the plot we do not display the value of $\omega _0$ approximately equal to $1$ even after the global phase.

We can draw the following conclusion from these experiments. The global phase has found some points with $\omega _2$ parameter ranging from 20 to 100. However, only points with $\omega _2$ approximately equal to 20 have minimal misfit and the local phase has corrected them slightly.

### Global “floating-point” search

We have also implemented and executed the HGS algorithm with a floating point coding. The HGS used the same three accuracy levels as in the binary case and the same strategy was used for selecting local phase starting points. Also the population sizes, mutation and crossing rates were as in the binary case. The scale parameters utilized by the floating point search are the following:

$$\begin{aligned} \eta _1 = 16384, \ \eta _2 = 128, \ \eta _3 = 1 \end{aligned}$$

The floating-point HGS parameters are summarized in Table 3.

**Table 3:** Parameters of the floating point HGS

	Root	Intermediate level	Leaves
Population size	12	6	4
Mutation probability	0.1	0.01	0.001
Mutation std. dev.	1.0	0.2	0.01
Crossover probability	0.5	0.5	0.5
Crossover mean	0.5	0.5	0.5
Crossover std. dev.	0.01	0.01	0.01
Sprout std. dev.	0.1	0.01	
Sprout min. distance	0.5	0.2	
Sprout max. value	2	0.5	
Encoding scale ($\eta$)	16384	128	1
Ratio	265	13557	694136

**Fig. 7:**
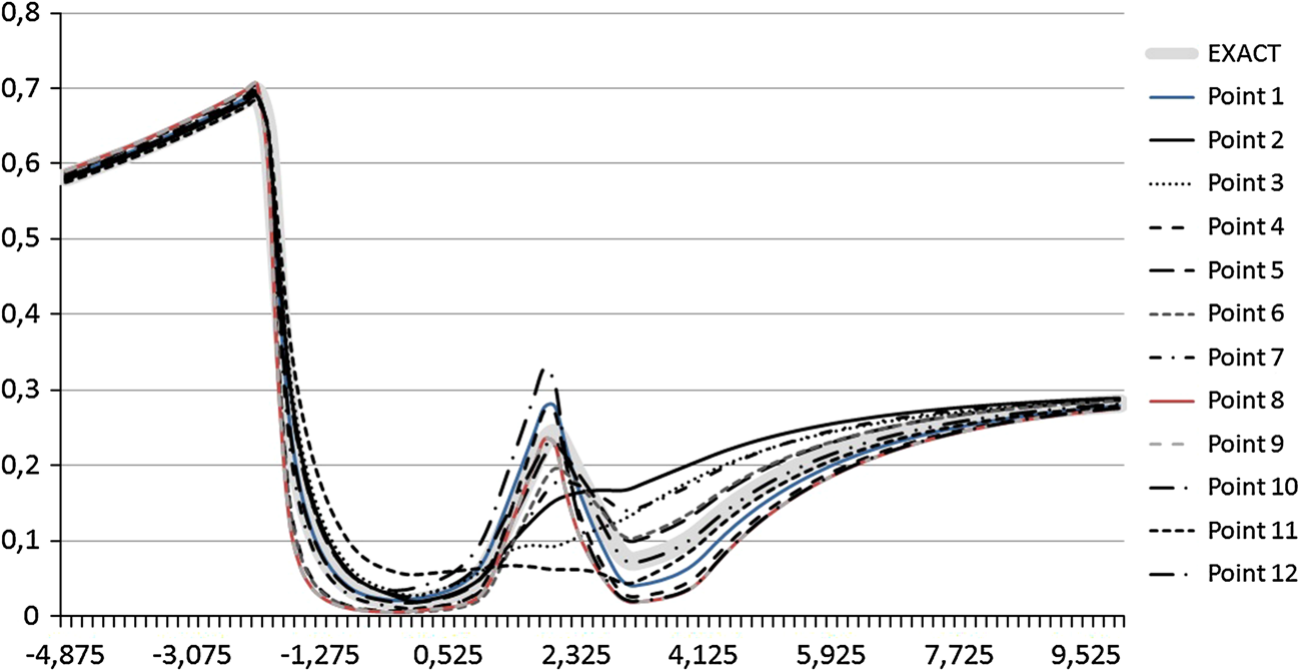
The *logging curves* corresponding to points found after floating-point global search phase. The *bold gray curve* corresponds to the exact *logging curve*

**Fig. 8:**
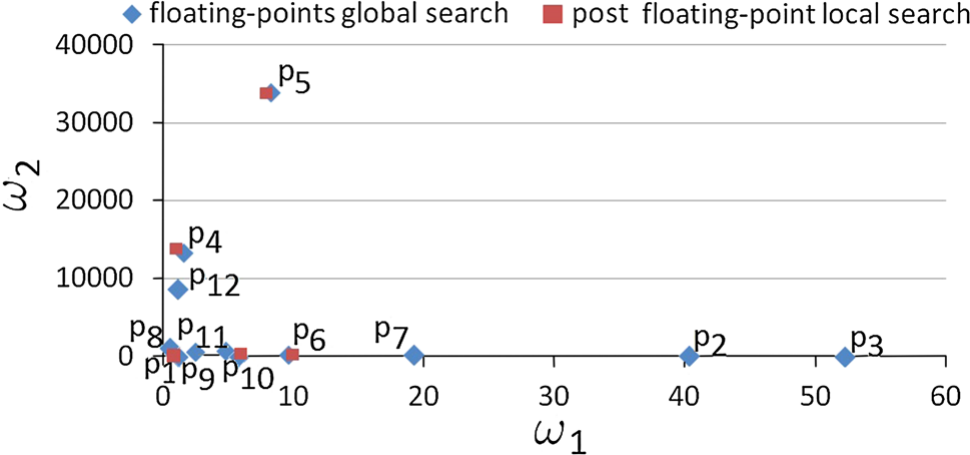
The results of the global and local floating-point phases. The particular points are denoted by $P_1,....,P_{12}$ labels, since they correspond to the twelve curves presented in Fig. 7

The floating point HGS algorithm found the following twelve starting points, summarized in Table 4. Again, for testing purposes, we have executed the self-adaptive goal-oriented $hp$–FEM algorithm on these points, in order to generate and plot the resulting logging curves. The curves corresponding to the found twelve points are presented in Figurepost-binary-curvespost-binary-curves Fig. 7. The curves have been also compared to the exact logging curve, denoted by bold light gray color. We can see that the floating-point global search has found much more points than binary global search and generally more points are better fitted. The twelve points obtained after the global floating-point phase are also depicted in Fig. 8 by twelve diamonds. The figure does not present the values of $\omega _0$ since they are all approximately equal to $1$.

**Table 4:** Results of the floating point global search

	$\omega _0$	$\omega _1$	$\omega _2$	*Misfit*
Point 1	1.003	2.287	491.275	0.0309955725861
Point 2	1.518	40.215	81.736	0.18913950464
Point 3	1.677	52.161	15.336	0.18563908906
Point 4	0.429	1.441	13317.938	0.0998952955352
Point 5	0.955	7.895	33995.309	0.0155211450748
Point 6	0.410	9.422	409.705	0.0788856673271
Point 7	0.803	19.114	275.680	0.103517536713
Point 8	1.541	0.469	1089.258	0.234231539517
Point 9	0.320	1.090	40.410	0.158735090456
Point 10	1.436	5.081	64.404	0.0123941038654
Point 11	2.370	5.692	3.732	0.248640339227
Point 12	1.398	1.038	8691.385	0.13256144668

### Local “post-floating-point” search

The local phase was executed from the five best fitted points obtained from the floating-point global phase. In particular, we have executed the local phase from Point 1, Point 4, Point 5, Point 6 and Point 10, since their misfit values are less than $0.1$. We have utilized the BFGS method again, and the relative error of the self-adaptive $hp$–FEM algorithm was set to $0.001$. The results from the floating-point global and local phases are summarized in Fig. 8. The plot does not present the values of $\omega _0$ since they are approximately equal to $1$. The local phase has changed the locations of these five best fitted points slightly, which is denoted in Fig. 8 by squares.

This group of experiments entitles us to the following conclusions.

The floating-point global phase has found much more points than the binary global phase. In particular, the floating-point global phase has found some points with $\omega _1$ parameter ranging from 0 to 60, as well as $\omega _2$ parameter ranging from 3 up to 33000. However, only points with $\omega _1$ from range of 0 to 10 have minimal misfit and the local phase corrected them slightly.

## Comparison with state-of-the-art methods

The goal of this section is to provide a fair comparison of our hybrid $hp$–HGS strategy with classic state-of-the-art methods widely utilized in the continuous global optimization.

The first reference method is the Simple Evolutionary Algorithm (SEA) (see e.g., Törn (1975)), the second one is the multistart method (MS) (see e.g., Michalewicz (1996)) utilizing the BFGS (Broyden-Fletcher-Goldfarb-Shanno) quasi-Newton local optimization algorithm Nocedal and Wright (2006). The BFGS implementation was taken from the SciPy scientific Python library Scientific Computing Tools (2001). All above methods were executed for the DC inverse problem described in Sect. 9.

Let us first estimate the budget of the floating-point encoded $hp$–HGS hybrid strategy with BFGS local search, applied to the solution of the DC problem. The budget $T_{budget}$ is defined as the amount of time spent on solving the DC problem by the hybrid algorithm on a single workstation with quad cores, where all the calls of the self-adaptive $hp$–FEM were serial, but the $hp$–FEM code itself utilized four cores for each computation. The computational budget can be estimated by the following formula:

47 $$\begin{aligned} T_{budget} = t_{root}*N_{root} + t_{inter}*N_{inter} + t_{leaves}*N_{leaves} + t_{local}*N_{local}*N_{iter} \end{aligned}$$

where $t_{root}$ and $N_{root}$ are the average time of calling the self-adaptive $hp$–FEM with the accuracy of the root level and the number of such calls, respectively, $t_{inter}$ and $N_{inter}$ are the average time of calling the self-adaptive $hp$–FEM with the accuracy of the intermediate level and the number of such calls, respectively, $t_{leaves}$ and $N_{leaves}$ stand for the average time of calls of the self-adaptive $hp$–FEM with the accuracy of the level of the leaves and the number of such calls, respectively, $t_{local}$ is the average time of calling the self-adaptive $hp$–FEM with the accuracy of the local method, $N_{local}$ stands for the number of calls of the self-adaptive $hp$–FEM at the single iteration of the local method, and $N_{iter}$ is the average number of iterations of the local gradient method.

By analyzing the log files from the $hp$–FEM hybrid method execution, we have estimated the total budget $T_{budget}=8523.4$ minutes. We have also obtained the average execution time for the self-adaptive $hp$–FEM code for $4$ considered accuracies, $t_{root}=2.2$ minutes, $t_{inter}=2.7$ minutes, $t_{leaves}=10.0$ minutes, $t_{local} = 19.0$ minutes.

Next, we estimate the parameters of SEA and MS algorithms in such a way that the comparison of the three methods is fair. In particular, we provide each method with the same computational budget and assure the conditions under which they will work properly. We assume the relative error $0.001$ of self-adaptive $hp$–FEM calls utilized by both reference methods (SEA and MS), the same as used in the local method. In other words, within the computational budget $T_{budget}$ both methods can perform at most $T_{budget}/t_{local}$ $hp$–FEM calls.

Based on broad experience from the field of evolutionary computation, we can assume that the SEA algorithm should perform at least $t_{min\_epoch}=100$ epochs. It is necessary for enabling the evolutionary mechanisms to transform the initial population and converge towards misfit minima. From that estimate, it follows that the number of $hp$–FEM solver calls per genetic epoch in the SEA population should not exceed

48 $$\begin{aligned} \left\lceil \frac{T_{budget}}{t_{local}*t_{min\_epoch}}\right\rceil = \left\lceil \frac{8523.4}{19*100}\right\rceil = \left\lceil 4.486 \right\rceil . \end{aligned}$$

On the other hand, during a genetic epoch no less than 25 % of individuals evaluate their fitness (on the average), which results from the current parameter setting. Hence, we can safely assume the population size of 20.

In the MS search we used the BFGS algorithm, which estimates the gradient and the Hessian matrix in each iteration, which requires several calls of the self-adaptive $hp$–FEM code. Based on our experience obtained from the previous experiments, we can estimate the average number of calls for this method as $N_{BFGS\_calls}=20,$ and the average number of iterations required for the method to converge as $N_{BFGS\_iter}=5.$ Thus, the number of BFGS processes that can be successfully executed with the given budget can be estimated as

49 $$\begin{aligned} \frac{T_{budget}}{t_{local}*N_{BFGS\_calls}*N_{BFGS\_iter}}= \frac{8523.4}{19*20*5} \approx 4.5. \end{aligned}$$

We assumed that about half of the local gradient methods will not converge, thus we utilize 10 starting points in MS generated with the uniform distribution over the search domain.

### Simple evolutionary algorithm

For the first comparison we used the Simple Evolutionary Algorithm with the population size $20$. Other genetic parameters are summarized in Table 5. We used proportional selection and arithmetic crossover. The crossing rate is the probability of selecting a genetic individual for reproduction. Analogously, the mutation rate is the probability of selecting an individual for mutation. During mutation, a new (mutated) individual is sampled according to the normal distribution centered in its parent and with a given standard deviation. The offspring “born” outside the search domain is ignored, no repairing mechanisms were applied (see e.g., Michalewicz (1996) for details). The SEA algorithm has been executed for 15768 minutes, and it performed 138 genetic epochs with 714 self-adaptive $hp$–FEM solver calls, so the assumed budget $T_{budget}=8523.4$ minutes was exceeded. It approached only one minimum at $[1.7757, 1.3518, 10859.5794]$ with fitness value $0.1648$. All other individuals had either much larger fitnesses or were concentrated around the same local minimum.

**Table 5:** Parameters of SEA

Population size	20
Crossing rate	0.5
Mutation rate	0.01
Mutation standard deviation	0.01
Relative $hp$–FEM error	0.001

### Multi-start

The second method executed for the comparison was the multistart (see e.g., Törn (1975)), which ran the local BFGS search algorithm from 10 starting points sampled randomly (see Table 6). The BFGS algorithm stopped after $1$ or $2$ iterations for $5$ starting points because a very small gradient norm was encountered. For other $5$ points various numbers of iterations (from $6$ to $52$) were performed, however the value of the fitness function as well as the values of the parameters were not updated significantly. The total a posteriori execution time for the MS method was around 50123 minutes, which exceeds the assumed budget almost 6 times. BFGS started from Point 1 finished with the misfit value about $0.33$, the other BFGS runs ended with the misfit value greater than $3$, *i.e.* no satisfactory local minimum was encountered.

**Table 6:** Points for the multi-start method together with number of iterations and solver calls for BFGS algorithm

	$\omega _0$	$\omega _1$	$\omega _2$	*Iterations *	*Calls *
Point 1	0.776	150.412	463.757	2	41
Point 2	79102.653	0.134	30.726	52	1483
Point 3	852.585	1623.620	74793.589	13	278
Point 4	10834.989	12.119	2331.844	7	148
Point 5	38.572	1.800	570.589	1	5
Point 6	665.296	938.495	0.171	1	5
Point 7	47.764	32843.280	0.426	1	5
Point 8	76554.300	312.651	25.982	6	123
Point 9	68.738	2136.115	32781.281	9	175
Point 10	25805.765	2.959	2.598	1	5

## Conclusions

We described a hybrid strategy for solving inverse problems exhibiting multiple minima. The strategy utilized a $hp$–HGS algorithm on the global level.

The $hp$–HGS algorithm tuned the length of the genetic code (or the accuracy of representation in case of floating-point implementation) as well as the accuracy of the goal-oriented self-adaptive $hp$–FEM solver. This allowed to find relatively quickly on the global level the regions where we expect to find the local minima. In these regions, we increased the accuracy of the genetic search by increasing the length of the genetic code (or the accuracy of representation in case of floating-point implementation) and the requested accuracy for the goal-oriented self-adaptive $hp$–FEM solver.

After several iterations of the $hp$–HGS strategy, we switched to the (local) gradient search to converge quickly to the local minima within the regions delivered by the $hp$–HGS algorithm.

The $hp$–HGS strategy requires knowledge of the relation of approximate forward and inverse solutions errors. We estimated the error relation for the considered problems in Sect. 6 (see Propositions 2, 3 and Remark 5). The crucial features necessary for establishing this relation were Lipschitz continuity of the primal solution with respect to the conductivity distribution (see Lemma 1) and the proper evaluation of the quantity of interest functional taken for the relative $hp$–FEM error (see Lemma 2).

We tested our strategy on a challenging numerical problem consisting of the inversion of 3D DC borehole resistivity measurements. In the global phase we performed tests for $hp$–HGS with both binary and floating-point encoding. The binary $hp$–HGS found only six starting points, whereas the floating-point $hp$–HGS found twelve starting points, proving to be a more powerful tool for global phase computations. After the global phase, the found value of $\omega _0$ was approximately equal to 1 as expected, however the values of $\omega _1$ varied between (6.54,70.62) after the binary global search, and between (0.46, 52.16) after the floating-point global search. Similarly, the values of $\omega _2$ varied between (31.90, 99.77) after binary global search and (3.72, 33.99) after floating-point global search. We have selected the best fitted points after the global phases and executed the local gradient search with the BFGS method. The local phase check executed after the binary global search phase resulted in the final points $\omega _0\approx 1$, $\omega _1 \in \{5, 20\}$ and $\omega _2 \approx 40$ (compare Fig. 6). This may suggest that the problem is not very sensitive to the $\omega _1$ value. If we compare these results from the results of the local phase executed after the global floating-point search, we can see that the final points obtained from the execution of the latter have the following properties: $\omega _0 \approx 1$, $\omega _1 \in (0; 10)$, and the problem is insensitive to $\omega _2$ value (compare Fig. 7). In order the verify our conclusion that the problem is insensitive to $\omega _2$, we executed the self-adaptive $hp$–FEM algorithm for all the points found after the global floating-point search. We plotted the corresponding logging curves in Fig. 7, and compared them to the exact logging curve. The comparison showed that points with low fitnesses actually have almost identical logging curves that cannot be distinguished by the inverse problem solver. We may conclude that the floating-point coding algorithm allows to find additional results that cannot be found by binary encoding algorithm. We can also claim that DC measurements are not sensitive to the resistivity of $\omega _2$.

These results enable an expert on the field to evaluate all possible solutions, and thus, they allow to better estimate the subsurface properties as well as to assess the uncertainty level. Thus, the proposed hybrid method provides an adequate alternative for solving challenging multimodal inverse problems.

The comparison of the method with state-of-the-art methods like Simple Genetic Algorithm or the Multi-Start method shows that only the hybrid $hp$–HGS can satisfactory explore the landscape of the inverse problem solution under the assumed computational budget, while SEA method converged only to one local minimum and the MS method got stuck on areas with the gradient being approximately zero for all the selected points.
